# Acoustocerebrography in septic patients: A randomized and controlled pilot study

**DOI:** 10.3389/fmedt.2022.920674

**Published:** 2022-09-14

**Authors:** Martin Sauer, Anika Sievert, Miroslaw Wrobel, Paul Schmude, Georg Richter

**Affiliations:** ^1^ Department of Anesthesiology and Intensive Care Medicine, University Hospital Rostock, Rostock, Germany; ^2^ Fraunhofer Institute Cell Therapy and Immunology, Leipzig, Germany; ^3^ Department of Intensive Care and Emergency Medicine, Hospital Magdeburg, Magdeburg, Germany

**Keywords:** critical illness, delirium, encephalopathy, molecular acoustic, severe sepsis

## Abstract

Sepsis-associated encephalopathy (SAE) is a common organ dysfunction in patients with severe sepsis or septic shock and leads to higher mortality and longer hospital stay. The diagnosis remains an exclusion process; none of the available measurements are specific for SAE. The aim of the presented prospective and controlled clinical study was to evaluate the possible role of molecular acoustics in determining acute brain injury in septic patients using an acoustocerebrography (ACG) system. ACG is a multifrequency, transcranial ultrasound method that measures the attenuation and time of flight to detect changes in the brain tissue. After approval from the local research ethics committee (of the University Hospital of Rostock: Reg. No.: A 2016-0026), 20 patients were included in two study groups: septic shock group (SG) and control group (CG; postoperative nonseptic patients). All patients were screened several times with the ACG on different days. Blood parameters of organ function, sepsis-related organ failure assessment score, and delirium scores [Confusion Assessment Method for the Intensive Care Unit (CAM-ICU) and Intensive Care Delirium Screening Checklist (ICDSC)] were obtained as well. A neurologist examined all patients at inclusion. Predictive analysis was done using a data-driven statistical method and by deriving a parameter from the ACG data. The study was registered under “clinicaltrials.gov” (Reg. No.: NCT03173196). All patients in the SG were CAM-ICU-positive at inclusion (ICDSC: in mean 4.0) and had clinical signs of SAE. In contrast, all patients in the CG were CAM-ICU-negative, with an ICDSC score of 0. Predictive analysis using the ACG data presented an accuracy of 83.4% with a specificity of 89.0% and a sensitivity of 75.1%. The ACG method may be helpful for the monitoring and diagnosing acute brain injury; however, the results of this first report should be verified by further clinical studies. Further investigations should include long-established instruments of SAE diagnosis, e.g., electroencephalography, MRI, and biomarkers, to compare the results with the ACG measurements.

## Introduction

The incidence and mortality of sepsis are high and remain stable despite some advances in supportive therapy (1–3). Additionally, the sepsis-associated encephalopathy (SAE) occurred in nearly 70% of patients with severe sepsis or septic shock and may lead to acute brain injury (4). The multifactorial syndrome SAE is a reversible brain dysfunction induced by the systemic response to the infection without evidence of direct brain infection and is associated with a high mortality rate (4, 5). Despite all research efforts, the knowledge of the underlying mechanisms of SAE is low and more studies have to be performed to get insight into this important field (4–7). Clinical presentations of SAE range from mild symptoms to severe agitation or deep coma, with increasing evidence for long-term physical, cognitive, and psychological impairment in sepsis survivors (4, 8). Common screening tools for SAE are, for instance, the Confusion Assessment Method for the Intensive Care Unit (CAM-ICU) and the Intensive Care Delirium Screening Checklist (ICDSC) (4). In patients with SAE, abnormalities were found in electroencephalography (EEG), somatosensory-evoked potentials, and neuroimaging (4, 5, 9–12). Additionally, an increase in biomarkers such as neuron-specific enolase and S100 beta protein was seen (11, 13). None of these abnormalities are specific to SAE, and the diagnosis remains an exclusion process (4, 9–11). All diagnostic tools for the exclusion diagnosis SAE are time- and cost-consuming. Therefore, it is of high clinical interest to diagnose SAE as early as possible as it is crucial for neurological outcomes (4, 6–8).

The aim of the presented pilot study was to evaluate the possible role of molecular acoustics in determining brain injury in patients with septic shock. Using an acoustocerebrography (ACG) system, a prospective and controlled pilot studyonpatients was conducted.

**Figure 1:**
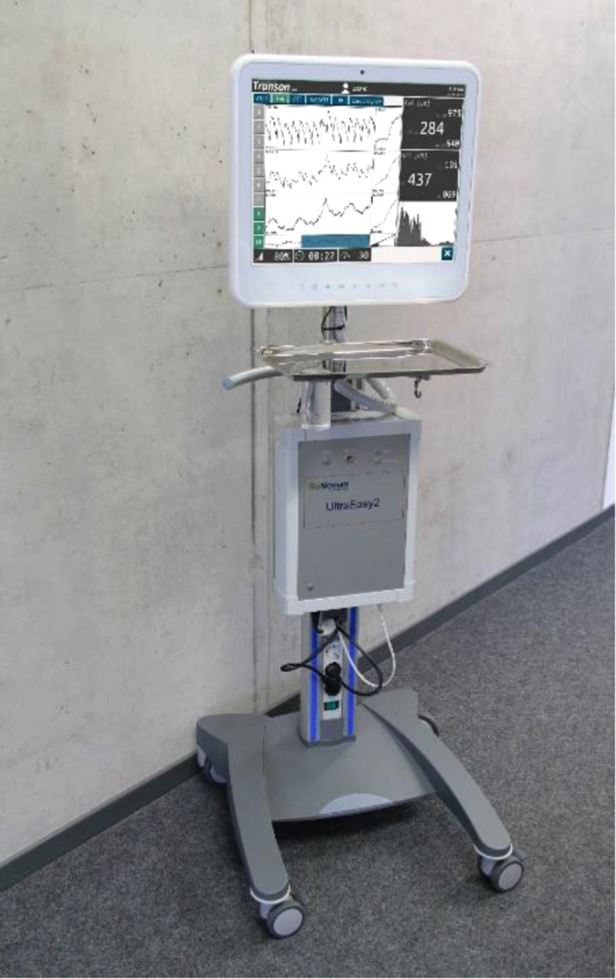
UltraEASY2 system used in the study. All components are assembled into a trolley to be transportable. The software component (Transon 2.0) does not give an analysis and is only used for data acquisition.

The ACG device (Figure 1) used in the study was approved for clinical trials (according to EN 60601-1, EN 60601-2-37, and EN 55011), and it consists of two ACG probes joined by a strap on the patient’s head connected to an output device with a touchscreen that displays ACG measurement data.

In this paper, we present a new brain diagnostic method based on a computer-aided multispectral ultrasound diagnostics method (14–17). In one of the first in-man studies with healthy volunteers, we explored the standard values of the relative time of flight (TOF) and the attenuation (ATT) of multispectral longitudinal ultrasound waves propagated noninvasively through the brain across patients of different ages and genders. For interpretation of the volunteer's health questionnaire and ultrasound data, we explored various clustering and classification algorithms, such as PCA and ANOVA. We showed that the TOF and ATT values provide very good estimation of possible physiological changes in the brain tissue and can differentiate the possible high-risk groups obtained by other groups and methods (18). The same approach, using advanced mathematics to calculate the measured values, is presented in this paper.

## Material and methods

### Subjects

Twenty patients from two perioperative intensive care units (ICUs) of the University Hospital Rostock were included in the study. Written informed consent was obtained from all patients or from the patients’ representatives if direct consent could not be received. The study was registered under “clinicaltrials.gov” (Reg. No.: NCT03173196), conducted in accordance with the Declaration of Helsinki, and received ethics approval from the local research ethics committee (Ethical Committee of the University Hospital of Rostock, Reg. No.: A 2016-0026).

Between June 2016 and June 2017, all patients in the ICU with age over 18 years were screened for the parameters of severe sepsis or septic shock as defined by international consensus criteria [Sepsis-1 criteria (19)] and ten surgical patients with septic shock were included in the septic group (SG). Exclusion criteria included lack of informed consent, other causes for delirium, predescribed cerebral alteration, and beginning of sepsis longer than 24 h. At inclusion, a neurologist examined all patients. The control group (CG) included ten postoperative patients after abdominal surgery without signs of sepsis and delirium, without predescribed cerebral alteration, and an estimated ICU stay longer than 24 h.

### Study procedures

Patients were monitored for 28 days (observation time), and hospital survival, premorbidity, basic demographic information, illness severity [acute physiology and chronic health evaluation (APACHE II), sepsis-related organ failure assessment (SOFA) scores], microbiological results, and clinical outcome for study cohort were recorded. On days 1, 3, 7, 14, and 28 (in the CG, only on days 1, 3, and 28), the patients were screened for clinical data: hemodynamic, use of norepinephrine, inflammation, coagulation, temperature, and organ function blood parameters; the SOFA score and delirium scores (CAM-ICU and ICDSC) were obtained at the same time points.

### Acoustocerebrography

**Figure 2:**
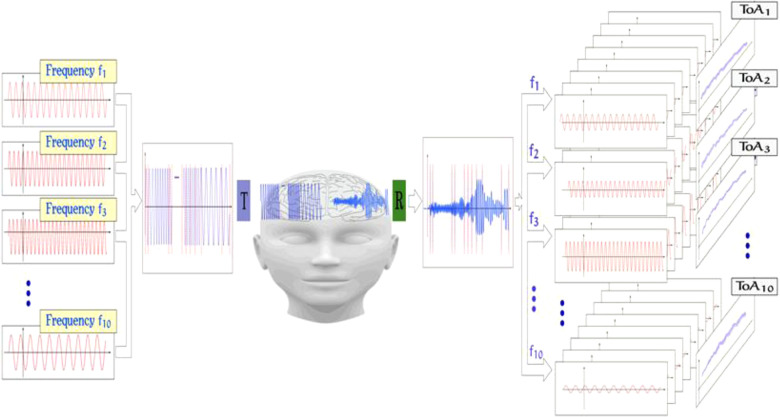
Signal acquisition method of the ACG; from left to right: different frequency components in the range of 0.5–2.5** **MHz are chosen, and an input signal is formed by either concatenating the frequency packages or by superposition of those. The signal is then emitted from an ultrasound transducer and propagated through the brain. The complex ultrasonic signal is then received and sampled on the other side of the skull. By comparing both input and output signals, features like, for example, TOF and signal ATT are extracted.

ACG is a noninvasive, transcranial ultrasound method with the purpose of measuring the elasticity and density of the brain tissue (20–22). It utilizes the research results of molecular acoustics, referring to the science of transmission mechanisms of acoustic energy *via* molecules in liquids and gases. Due to Kramers–Kronig relations, the velocity of ultrasound depends significantly on the frequency of investigated waves (23). In nonlinear material, like human brain tissue, an effect of longitudinal wave dispersion can be clearly observed and measured. The nonlinear, frequency-dependent bulk modulus of the medium results in different propagation speeds for different ultrasound frequencies. In addition to the observed changes in propagation speed, different attenuation profiles can also be observed. The mechanism is further explained in Figure 2.

### Measurement with acoustocerebrography

Patients in the CG were monitored by ACG (UltraEASY2, Sonovum, Leipzig, Germany) twice, at inclusion and after 3 days, and the patients in the SG were monitored at inclusion and after 1, 3, 7, and 14 days. The measurement procedure lasted 30 min, partitioned into 10 single measurements. Every measurement consisted of placing the probes on the head (on both temporal sides), applying ultrasound gel, followed by the measurement itself. Several measurements were done to eliminate effects regarding the exact positioning of the probes.

**Figure 3:**
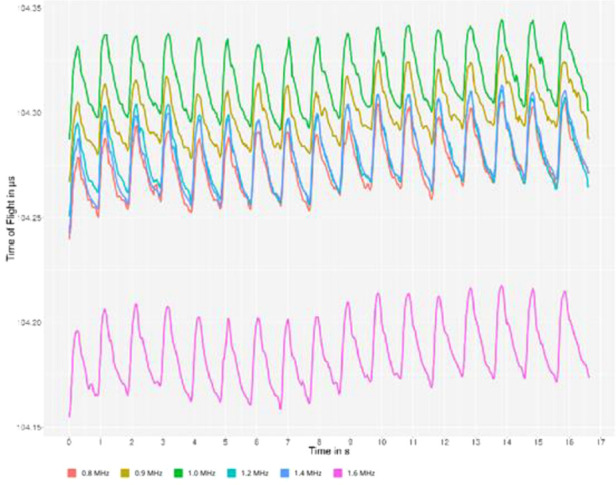
Typical multispectral ultrasonic curves can be observed on the monitor of the device during the examination. The *x*-axis represents the timescale, and the *y*-axis represents the time difference between sending and receiving the ultrasound signal of a specific frequency, called time of flight. The color codes represent different frequencies of ultrasound.

Using multiple frequencies shows the dispersive character of brain tissue and provides new interpretations for signal changes. In our study, due to the very frequency-selective method provided by ACG, we can measure both parameters of tissue dispersity in the form of TOF and selective ATT. TOF shows increases in the stiffness of brain tissue, which is directly linked to the change in the bulk modulus. In ATT, some small scattering changes in the cellular brain system play a dominant role but not the absorption of the ultrasonic wave. An example of such measurements can be found in Figure 3.

### Data processing

The data were processed using two different approaches. The first is a deterministic one, where a parameter based on theoretical assumptions was created, referred to as the dimensionality of the data. The second one is a stochastic one, which involves genetic algorithm (GA) feature selection and principal component analysis.

To detect maleficent changes in the brain, a feature called dimensionality of the data is proposed, which is a measure for asynchronicity of frequency-related features. In a healthy brain, frequency-related features should be almost in sync, while a brain disorder lowers the synchronicity and, therefore, increases the dimensionality of the data (18, 21, 24, 25).

To compute this feature, at least two frequency-dependent time series, in this case, the TOF or ATT, for 10 different frequencies are used. For each data matrix, the principal component analysis (a singular value decomposition of the data matrix) is computed, and especially the explained variance for each component is of interest. The dimensionality of the data is defined as the minimal number of components necessary to reach a threshold of explained variance; for example, how many principal components are necessary to explain 95% of the variance of the system. This imitates the value of the dimensionality of the data from 1, meaning that almost all of the dynamics of the system can be described by using only one linear combination of the input values, to the number of frequencies used, which means that the value shows a different dynamic for every frequency (20, 21).

**Figure 4:**
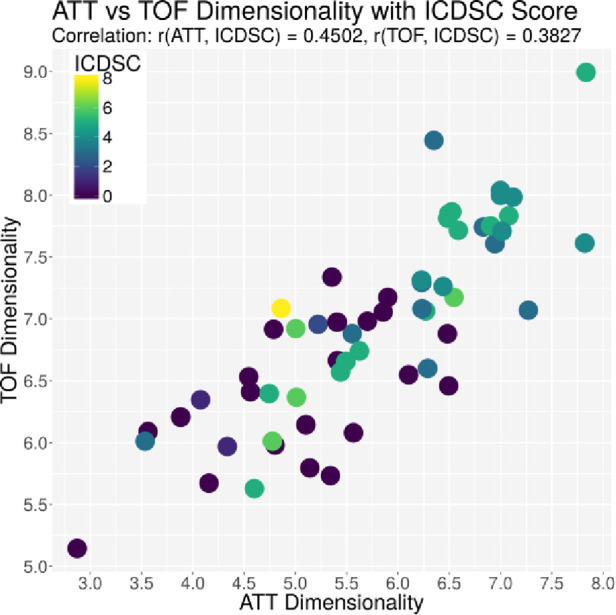
Plot of the dimensionality of the ATT vs. the dimensionality of the TOF. Every dot represents one patient on day 1; the color represents the ICDSC score of that patient on that day.

As another way to analyze the data, we use a genetic algorithm to detect covariates with the target variables. The genetic algorithm feature selection is based on the nature concept of evolution. It was first adapted by Barricelli (26, 27), and Holland (28) made the concept widely known in 1975. The algorithm contains the steps for creating a population, assessing the fitness of every individual, mating of the fittest, and mutation. Each phase will be briefly explained, including the specific adaptations for the present case, as can be seen in Figure 4. It has been shown by Schmude (29) that GA feature selection is a viable option in the context of ACG data.

An individual of the population is a subset of features, and the features are restricted to a maximum of 15 (out of over 1,000). As initialization, 1,000 randomly selected feature subsets are created.

The fitness of every individual is evaluated by analyzing the feature subset as the next step. As a target function, the principal components of the data matrix are obtained from the 15 features of the individual. This unsupervised technique is used to limit the possibilities of combining the features and to target overfitting. The first principal component, the one that explains the highest amount of variance, is then correlated with the targeted medical parameter. The aim of this approach was to find a high absolute correlation as a measure of fitness.

After evaluation of the current generation, the following generation is built. Individuals with the highest fitness (top 2%) are combined to create new individuals. Two individuals are combined by combining their features; if they have a feature in common, the feature stays in their child; if they both do not share a feature, it will not be seen in the child; if one of the parents has a feature that the other one does not, the feature will be randomly added or dismissed with a 50% chance. The rest of the population is filled up with randomly created individuals.

To avoid being stuck in evolution, the mutation of the child in the new generation as the last step is obligate. By randomly deleting and adding features to the individuals, changes in the population are enforced, even if the top individuals are similar. This step is also used to check if every individual has a maximum of 15 features. Otherwise, surplus features will be randomly deleted.

The above-mentioned steps, except the initialization, describe one generation. The process of fitness evaluation, combination, and mutation was repeated for 200 generations until no significant improvement in terms of the fitness function of the best individual appeared.

### Statistics

The results of patient data are expressed as the median with 0.25–0.75 quartile. The results of the study are not available in a normal distribution, and statistical differences between groups are analyzed using the Kruskal–Wallis test followed by the Mann–Whitney *U* test for pairwise comparison using the Statistical Package for the Social Sciences (SPSS, Chicago, USA). The chi-squared test was used for the comparison between both groups for the CAM-ICU results.

The results of ACG measurements are expressed as the average value of a patient on a particular day. R [R Core Team (2016). R: A language and environment for statistical computing, Vienna, Austria. www.r-project.org] was used to process the data and to calculate the Pearson correlation between the medical parameters and the features derived from the ACG measurements. Due to the exploratory character of the study, combined with an overload of features and a comparatively small number of individuals, normal statistical methods would not give reliable results. The data processing focused on finding features and combinations of features that could possibly have predictive power. Therefore, the two methods (genetic algorithm, dimensionality) were preferred over the usual statistical methods.

Differences and correlations were considered significant at *p* < 0.05.

## Results

### Disease severity, survival, and results of laboratory parameters

One out of the 10 patients of the SG died on day 26. All other patients of both groups survived the hospital stay. All patients of the SG fulfilled the criteria for septic shock at inclusion. There were two female patients in each group; the median age was 65 years in both groups. Table 1 shows an overview of SOFA and APACHE II scores and laboratory parameters on days 1 and 3 of the patients in the SG compared with those in the nonseptic control group (CG). Significant differences between both groups were seen in all scores, the number of leukocytes, partial thromboplastin time, creatinine, and urea. Four patients in the SG and only one in the CG developed a severe kidney injury; renal replacement therapy (continuous methods) was necessary for three patients in the SG. In addition, two patients fulfilled the criteria of liver dysfunction in the SG at inclusion; however, on day 7, the liver function of these patients was normalized.

**Table 1:** Results of laboratory parameters, partial oxygen pressure, and scores on days 1 and 3 in the CG and the SG.

Parameters	Day 1			Day 3		
	CG (*n* = 10)	SG (*n* = 10)	*p*	CG (*n* = 10)	SG (*n* = 10)	*p*
Leukocytes (10^9^/L)	8.9 (7.2/10.8)	10.6 (8.0/14.4)	n.s.	8.4 (6.1/10.2)	12.3 (10.0/14.7)	0.045
Platelets (10^9^/L)	152 (121/176)	205 (179/268)	0.013	134 (123/153)	162 (125/213)	n.s.
Quick (%)	85 (80/92)	64 (58/77)	0.017	96 (86/103)	92 (84/93)	n.s.
PTT (s)	29 (27/31)	40 (38/45)	<0.001	30 (29/31)	37 (32/40)	0.019
ALAT (U/L)	23 (17/36)	23 (1d7/47)	n.s.	27 (20/37)	31 (24/56)	n.s.
ASAT (U/L)	21 (16/56)	34 (30/66)	n.s.	25 (16/39)	63 (29/127)	n.s.
Bilirubin (µmol/L)	12 (8/15)	21 (13/38)	n.s.	6 (0/14)	19 (17/22)	n.s.
Creatinine (µmol/L)	83 (72/90)	134 (103/200)	0.013	73 (66/88)	100 (89/182)	0.028
CRP (mg/L)	n.d.	381 (320/342) (*n* = 4)	n.d.	101 (57.7/150) (*n* = 4)	319 (143/374) (*n* = 6)	n.s.
PCT (ng/ml)	n.d.	14.7 (7.4/34.6)	n.d.	0.5 (0.5/0.5) (*n* = 2)	9.4 (4.6/35)	0.032
Urea (mmol/L)	4.5 (3.8/5.4)	10.8 (7.1/11.9)	0.003	4.8 (4.1/6.2)	13.2 (7.7/16)	0.005
Lactate (mmol/L)	1 (0.7/1.1)	2.6 (1.4/3.1)	0.003	0.8 (0.8/1)	1 (0.7/1.2)	n.s.
PaO_2_ (kPa)	19.6 (14.6/20.2)	14.2 (11.2/16.3)	n.s.	12.4 (9.4/1566)	16.8 (12.4/19.2)	n.s.
pH (mol/L)	7.395 (7.375/7.41)	7.355 (7.3425/7.375)	0.028	7.42 (7.415/7.4325) (*n* = 4)	7.42 (7.3625/7.46)	n.s.
MAP (mmHg)	76 (71.75/78.5)	75.5 (72/84)	n.s.	83 (77/90)	78.5 (74.5/83)	n.s.
SAP (mmHg)	113.5 (110.25/116.5)	118 (114.75/122)	n.s.	120 (110/130)	122 (110.75/125.75)	n.s.
DAP (mmHg)	56.50 (52.5/62)	55 (52.25/63)	n.s.	60 (60/70)	58 (56/60)	n.s.
APACHE II (at ICU arrival)	9.5 (7.3/11.2)	26 (26/30)	<0.001	—	—	—
SOFA	3 (1.3/4)	12 (11.3/14)	<0.001	2 (0.3/2)	11 (6.3/13.8)	<0.001
CAM-ICU (positive)	0/10 patients	10/10 patients	<0.001	0/10 patients	9/10 patients	<0.001
ICDSC	0 (0/0)	4 (4/5)	<0.001	0 (0/0)	4,5 (3/5)	<0.001

ALAT, alanine aminotransferase; APACHE, acute physiology and chronic health evaluation; ASAT, aspatate aminotransferase; CAM-ICU, confusion assessment method for the intensive care unit; ICDSC, intensive care delirium screening checklist; n.s., not (statistically) significant; PaO_2_, partial arterial oxygen pressure; PTT, partial thromboplastin time; Quick, thromboplastin time; SOFA, sepsis-related organ failure assessment; CRP, C-reactive protein; PCT, procalcitonin; n.d., not done; CG, control group; SG, septic group; MAP, mean arterial pressure; SAP, systolic arterial pressure; DAP, diastolic arterial pressure. ICU, intensive care unit.

Median: 0.25–0.75 quartile.

### Delirium scoring in patients

The patients in the CG were screened with the CAM-ICU and ICDSC for delirium twice, at inclusion and after 3 days. The patients in the SG were screened at inclusion and after 1, 3, 7, and 14 days simultaneously to the ACG measurements (see Table 1). All patients in the CG were CAM-ICU-negative, and the ICDSC values were zero for all time points. In the SG, all patients were CAM-ICU-positive at inclusion; on day 3, nine patients were positive (day 7: 8 patients were positive, day 14: 7 patients were positive). The median values of the ICDSC scores in the SG were 4.0 at inclusion, 4.5 on day 3, 4.0 on day 7, and 5.0 on day 14. All patients had Richmond agitation sedation scale (RASS) values between −3 and +3 at the times of measurements. All patients of the SG had clinical signs of SAE, according to clinical examination by a neurologist.

### ACG results

**Figure 5:**
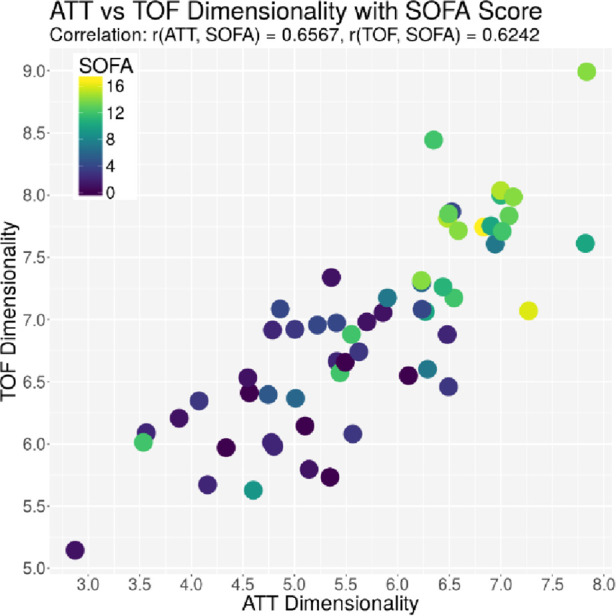
Plot of the dimensionality of the ATT vs. the dimensionality of the TOF. Every dot represents one patient on day 1; the color represents the SOFA score of that patient on that day.

**Figure 6:**
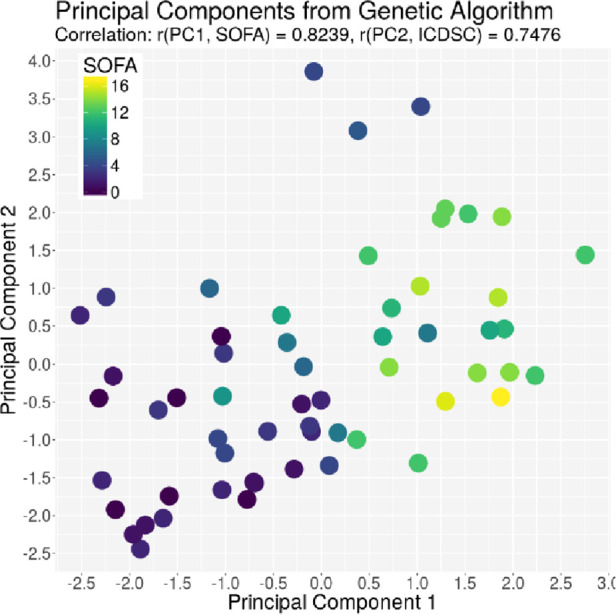
Plot of the principal component derived by the GA based on the SOFA score on the *x*-axis and the principal component derived by the GA based on the ICDSC on the *y*-axis. Every dot represents one patient on day1, color-coded with the SOFA index on that day.

**Figure 7:**
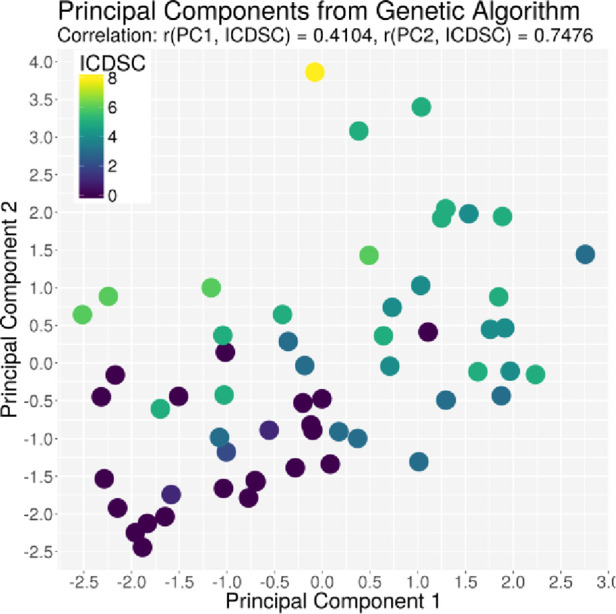
Plot of the principal component derived by the GA based on the ICDSC score on the *x*-axis and the principal component derived by the GA based on the ICDSC on the *y*-axis. Every dot represents one patient on day 1, color-coded with the ICDSC index on that day.

The ACG data were recorded with an average signal strength of 80.9% ± 14.6%, which is sufficiently high. As shown in Table 2, the best correlation of a medical parameter with the dimensionality is the SOFA score. The same is true for the genetic algorithm, which achieves its highest correlation with the SOFA score as well. Figures 5–7 visualize this correlation. It is possible to distinguish the two patient groups with their data, but in the case of the genetic algorithm, the separation is clearer. Furthermore, the ICDSC score and the levels of lactate, procalcitonin (PCT), creatinine, and bilirubin showed high correlations with the ACG data analyzed with the genetic algorithm (Table 2).

**Table 2:** Comparison of Pearson correlations of the dimensionality parameter and the principal components derived by the genetic algorithm with medical parameters.

Parameter	Correlation coefficient based on	
	Dimensionality	Genetic algorithm
SOFA	0.66	0.82
ICDSC	0.45	0.75
Lactate	0.32	0.75
PCT	0.17	0.82
Creatinine	0.13	0.70
Bilirubin	0.45	0.78

The genetic algorithm outperforms the dimensionality in all categories. The table shows the absolute correlation coefficients.

ICDSC, intensive care delirium screening checklist; PCT, procalcitonin; SOFA, sepsis-related organ failure assessment.

**Figure 8:**
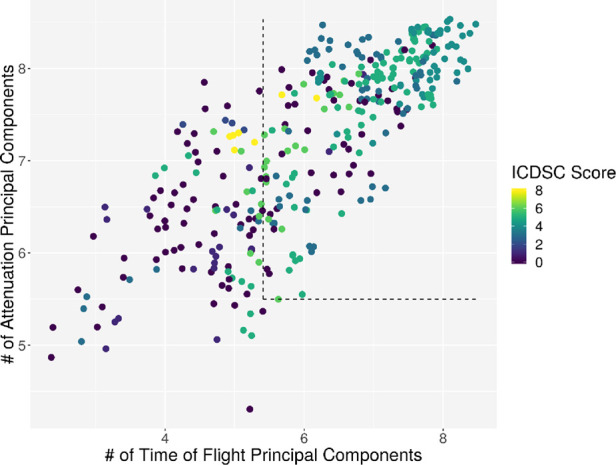
Scatterplot of the number of principal components necessary to explain 90% of the variance (dimensionality) calculated for 10 frequencies of attenuation. Each point represents a measurement of a patient at a given time; the points are colored according to their respective ICDSC score.

**Figure 9:**
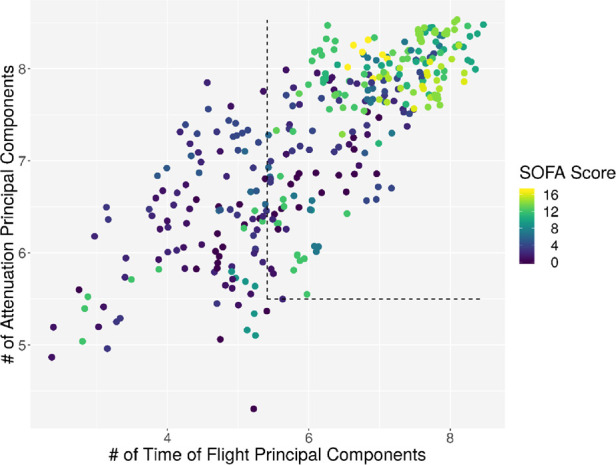
Scatterplot of the number of principal components necessary to explain 90% of the variance (dimensionality) calculated for 10 frequencies of attenuation. Each point represents a measurement of a patient at a given time; the points are colored according to their respective SOFA score.

**Figure 10:**
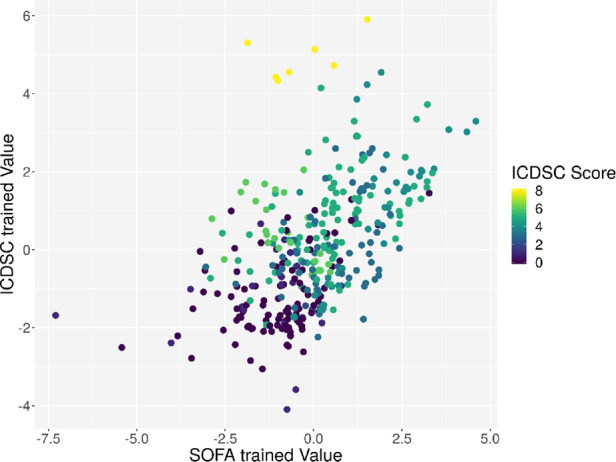
Scatterplot of the trained principal components. Each point represents a measurement of a patient at a given time; the points are colored according to the respective ICDSC score.

**Figure 11:**
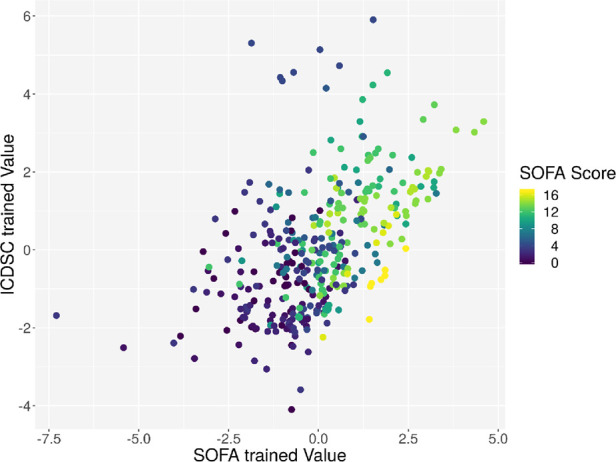
Scatterplot of the trained principal components. Each point represents a measurement of a patient at a given time; the points are colored according to the respective SOFA score.

Individual measurements were further analyzed. In Figure 8, the interaction between the ICDSC score and the dimensionality is plotted. A dotted line was drawn to suggest a separation between measurements where patients have high and low ICDSC scores. Figure 9 shows the relation between the SOFA score and the dimensionality. A dotted line was plotted to indicate a possible separation of patients with high and low SOFA scores. The features gained from the genetic algorithm were analyzed in Figures 10 and 11. Figure 10 shows the relation of the feature with the ICDSC score, and Figure 11 shows the relation with the SOFA score.

**Figure 12:**
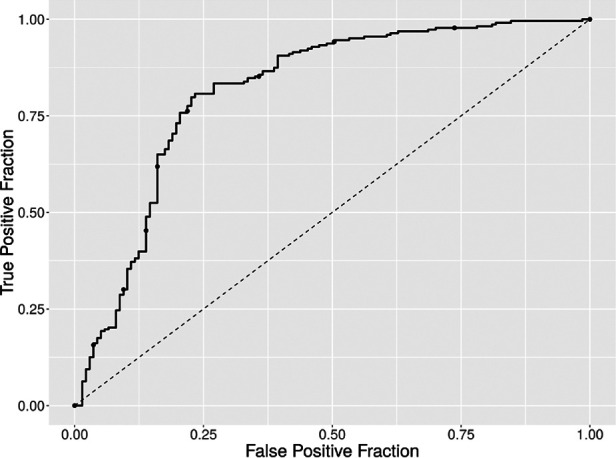
ROC curve of the binary classification of patients into two groups; group 1 with both a SOFA and an ICDSC score of 3 or more and group 2 containing all other measurements. The average accuracy was 83.39%, with an average sensitivity of 75.12% and an average specificity of 89.03%.

To determine the predictive power of the features gained, we divided the data into two subsets: patients with an ICDSC or SOFA score of 3 or more were labeled as positive; all other patients were labeled as negative. By using a support vector machine and fivefold cross-validation, an average accuracy of 83.39%, with an average sensitivity of 75.12% and an average specificity of 89.03%, was achieved. The receiver operator characteristic is plotted in Figure 12.

## Discussion

The pathophysiology of SAE is complex and, at present, not fully understood (4, 6). The known underlying mechanisms of SAE following septic inflammation are mitochondrial and endothelial dysfunction, blood–brain barrier dysfunction, neurotransmission disturbances, derangements of calcium homeostasis, and direct cellular damage in the brain tissue leading to acute brain injury (4–6, 30, 31). Brain magnetic resonance imaging can be normal or show brain edema and focal injuries, such as ischemic stroke or white matter hyperintensities (4, 5, 9, 10, 12). In the event, the development of SAE is associated with higher mortality and longer hospital stay (32).

The aim of this study was to evaluate the possible role of molecular acoustics in determining brain injury in septic patients using an acoustocerebrography system. The ACG system is a continuous, bedside applicable, and noninvasive monitoring of the brain tissue (20, 21).

The mortality in the SG was low with 10%, although higher values of APACHE II and SOFA scores of the patients in the SG were observed. All patients in the SG and CG were surgical patients for better comparability, and all patients in the SG had clinical signs of SAE, which is also displayed in the results of the CAM-ICU and ICDSC scores (Table 1) and the clinical examination of a neurologist. One limitation of this study is the lack of a more specific clinical examination for generating the exclusion diagnosis for “SAE.” Following investigations should include more classical instruments of SAE diagnosis, e.g., EEG, MRI, and biomarkers, to compare the results with the ACG measurement. Additionally, four patients developed an acute kidney injury (AKI) and two patients fulfilled the criteria of liver dysfunction in the SG at inclusion; however, on day 7, the liver function of these patients was normalized and three out of four patients with AKI were treated with continuous renal replacement therapy. The development of encephalopathy in study – patients may also cause by the two named organ dysfunction besides the causality of most likely SAE.

The exact diagnosis of SAE is a time-consuming exclusion process (4, 9–11); therefore, it is of high clinical interest to reduce the time of SAE detection affecting the central nervous system (4, 6–8).

In our study with the ACG system, as the first report in patients with severe sepsis and clinical signs of SAE, the dimensionality parameter reaches a good correlation with the SOFA score, which is surprising since the parameter was deterministically chosen based on theoretical assumptions. The dimensionality parameter itself is designed to be a measure of disorder in the brain, which might explain why no single laboratory parameter could be correlated with it. Since the dimensionality gives a measure for the system, like the SOFA score, it would be susceptible to many different parameters and their interactions rather than single parameters of organ failure.

The ICDSC, on the other hand, does not provide a good correlation with the dimensionality parameters. The state of delirium measured by the ICDSC can have various reasons and is, therefore, not a reliable source to show that the autoregulations in the brain are in disarray or do not fully display the inflammatory process in the brain during severe sepsis.

An interpretation of the results of the genetic algorithm can be made by taking into account the features that were picked to represent the SOFA and ICDSC scores. Both scores involve mainly a combination of attenuation-based values and features regarding the interactions between ATT and TOF. Interestingly, using the genetic algorithm, other clinical parameters regarding the severity of inflammation (PCT), microcirculatory impairment (lactate), and organ dysfunction (creatinine and bilirubin) showed good correlations with the ACG data also. A theory behind the change of ATT can be based on the edema caused by the SAE. The speed of sound of water at 37° is almost close to the speed of sound of brain tissue at that temperature (22), while the attenuation coefficient of brain tissue is much higher. That means a higher concentration of water in the probed field results in a similar TOF but a different ATT.

Both approaches yield respectable results, while the GA approach needs more data to learn; the dimensionality itself is a good indicator, although it should be considered alongside other parameters since it already produced some outliers.

The results of the classification algorithm utilizing dimensionality are promising but a little vague. The principal components found by the GA explaining SOFA and ICDSC were used with k-means clustering and a support vector machine to build a classification algorithm. The algorithm was trained using the first 10 patients and tested on the last 10 patients. The results show a correct prediction in five cases, while one case was wrongly predicted and the remaining four were undecided or varying. With more patients and further research, the classification of SAE and the understanding of the SAE interacting with ACG signals can be improved.

So far, ACG was only used to assess permanent damage to the brain; for example, Wrobel et al. (18) analyzed stroke risk factors in a large population, and Dobkowska-Chudon et al. (25) searched for an approximate number of white matter lesions in patients with atrial fibrillation. Both were able to find significant changes in the signals between the respective subgroups. Instead of detecting permanent brain damage, the current study focuses on dynamic changes in the brain tissue caused by SAE. The clinical symptoms of patients with severe sepsis and SAE change every day, and the ACG system can detect dynamic changes as well. Since the consequences of all the investigated disorders regarding the brain tissue are different, all studies used different analysis approaches but were based on the same system and the same measurement methodology.

Although no differences in blood pressure were observed between the septic and the control group on the measurement time points, hypotension, an atypical symptom in septic shock, may lead to a change in tissue characteristics. Looking at the physical parameters such as the speed of sound, sound absorption, density, and elasticity of biological tissue, it is immediately obvious that blood has the fastest speed of sound. At the same time, we know from other studies (33) that the change in blood pressure caused by the systolic/diastolic cycle causes a total skull expansion of about 20 μm for the systolic moment. In such a case, we have a superposition of two differently influencing two changing parameters. First, the decrease in TOF is due to the increase in the speed of sound with simultaneous expansion of the skull. In healthy patients, the system is very stable due to autoregulation. In our cases, the signals may be strongly influenced by the SAE, which we can show with this publication.

Lactate on day 1 was higher and pH was lower in the septic group than that in the control group (Table 1). Higher levels of lactate and lactate acidosis are well-described signs of microcirculation impairment, a typical symptom in septic patients. The impairment of microcirculation in the brain led to hypoxia and cerebral edema (5, 6). With cerebral edema, that is, swelling (edema) of the brain as a result of an increase in brain volume and pressure of various genesis, the liquor usually “gains the upper hand,” causing a decrease in the speed of sound and leading to an almost maximum extension of the skull. The coincidence of such parameters leads to a sharp increase in TOF and increased absorption of higher frequencies. In the worst case, cerebral circulatory arrest and complete signal loss can occur (33). The influence of acidosis on the acoustocerebrography measurement is yet to be investigated.

**Figure 13:**
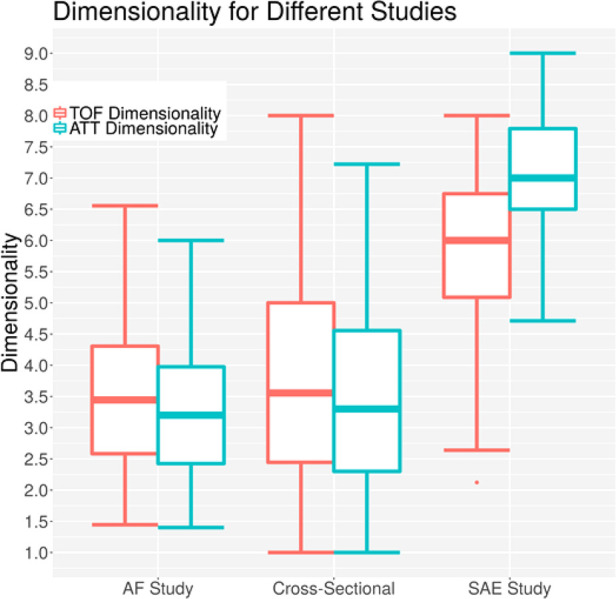
Dimensionalities for different acoustocerebrography-based studies; the *y*-axis is the dimensionality value, the *x*-axis represents the study, the TOF-based dimensionality is in red, and the ATT-based dimensionality is in blue.

Figure 13 shows a comparison of the data from different studies performed with ACG. The atrial fibrillation study had probands with no acute dysfunctions (25), and all were measured outside of a hospital with no symptoms occurring. Almost all patients had suffered a stroke at some point before, but none of them had an acute symptom. The study was performed to see if differences between the acoustic properties of their brain are different from probands without atrial fibrillation and to see if an estimate of white matter lesions based on the ACG signal is possible (25). The cross-sectional study included 294 patients with a large age range; all were measured in a normal state of mind without any acute conditions. The study was evaluated by Wrobel et al. (18). The average dimensionality values of both studies are lower than the dimensionality values of the current study (*p* value <1 × 10^−6^ for both studies), which is congruent with the explanation of the dimensionality parameter, which should only increase in acute phases of brain damage.

A limitation of the presented study is the small sample size and the impossible calculation of power for this approach in the discussion. The data of the presented pilot study should give us an idea of the power of differences between septic and nonseptic patients using acoustocerebrography. The dimensionality parameter may be the right choice for planning the sample size of further studies.

## Conclusion

The difficulties of diagnosing SAE remain, and a new, direct method is necessary. ACG may be used as a simple, quick, and noninvasive setup to give additional insights into the brain tissue. Further greater studies with this approach are needed for data validation of this first report with hopeful results. Following investigations should include more classical instruments of SAE diagnosis, e.g., EEG, MRI, and biomarkers, to compare the results with the ACG measurement.

## Data Availability

The raw data supporting the conclusions of this article will be made available by the authors without undue reservation.
